# Association of National Cancer Institute–Sponsored Clinical Trial Network Group Studies With Guideline Care and New Drug Indications

**DOI:** 10.1001/jamanetworkopen.2019.10593

**Published:** 2019-09-04

**Authors:** Joseph M. Unger, Van T. Nghiem, Dawn L. Hershman, Riha Vaidya, Michael LeBlanc, Charles D. Blanke

**Affiliations:** 1 SWOG Statistics and Data Management Center, Seattle, Washington; 2 Fred Hutchinson Cancer Research Center, Seattle, Washington; 3 Department of Epidemiology, Mailman School of Public Health, Columbia University Medical Center, New York, New York; 4 SWOG Group Chair’s Office, Knight Cancer Institute, Oregon Health & Science University, Portland

## Abstract

**Importance:**

National Cancer Institute Clinical Trial Network (NCTN) groups serve a vital role in identifying effective new antineoplastic regimens. However, the downstream clinical effect of their trials has not been systematically examined.

**Objective:**

To examine the association of NCTN trials with guideline care and new drug indications.

**Design, Setting, and Participants:**

This retrospective cohort study evaluated phase 3 SWOG Cancer Research Network clinical trials from January 1, 1980, through June 30, 2017. Only completed trials with published results were included. To be considered practice influential (PI), a trial must have been associated with guideline care through its inclusion in National Comprehensive Cancer Network (NCCN) clinical guidelines or US Food and Drug Administration (FDA) new drug approvals in favor of a recommended treatment. Data were analyzed from June 15, 2018, through March 29, 2019.

**Main Outcomes and Measures:**

Estimated overall rate of PI trials, as well as trends over time. The total federal investment supporting the set of trials was also determined.

**Results:**

In total, 182 trials consisting of 148 028 patients were studied. Eighty-two studies (45.1%; 95% CI, 37.7%-52.6%) were PI, of which 70 (38.5%) influenced NCCN guidelines, 6 (3.3%) influenced FDA new drug approvals, and 6 (3.3%) influenced both. The number of PI trials was 47 of 65 (72.3%) among those with positive findings and 35 of 117 (29.9%) among those with negative findings. Thus, 35 of 82 PI trials (42.7%) were based on studies with negative findings, with nearly half of these studies (17 of 35 [48.6%]) reaffirming standard of care compared with experimental therapy. The total federal investment spent in conducting the trials was $1.36 billion (2017 US dollars), a rate of $7.5 million per study or $16.6 million per PI trial.

**Conclusions and Relevance:**

Nearly half of all phase 3 trials by one of the NCTN’s largest groups were associated with guideline care or new drug indications, including those with positive and negative findings. Compared with the costs of a new drug approval in pharmaceutical companies, typically estimated at more than $1 billion, the amount of federal funds invested to provide this valuable evidence was modest. These results suggest that the NCTN program contributes clinically meaningful, cost-efficient evidence to guide patient care.

## Key Points

**Question:**

What proportion of National Cancer Institute–sponsored, phase 3 Clinical Trial Network program studies are associated with guideline care or new drug indications?

**Findings:**

In this cohort study based on 182 trials including 148 028 patients, 82 trials (45.1%) were associated with guideline care or new drug indications, including trials with positive and negative findings. The estimated federal investment for each practice-influential trial was $16.6 million.

**Meaning:**

The National Cancer Institute’s Clinical Trial Network program contributes clinically meaningful, cost-efficient evidence to guide patient care.

## Teaser

This cohort study examines the association of National Cancer Institute Clinical Trial Network studies with practice influence, measured as guideline care and new drug indications for treatment of patients with cancer.

## Introduction

The National Cancer Institute (NCI) Clinical Trial Network (NCTN) groups serve a vital role in identifying new cancer treatments. Unlike pharmaceutical companies—whose primary aims are to develop new drugs and generate profits—the mandate of the NCTN is to serve the community of patients with cancer more broadly. Network group trials may compare different treatment regimens, combine treatments and treatment modalities, and/or examine whether 1 or more newly approved drugs may work in other cancers.^1^ Although the network groups have been in existence since the 1950s, an examination of the clinical effects of network group trials as a research process has, to our knowledge, not been conducted to date.^2^

The mission of the NCTN groups is to “routinely achieve change in clinical practice” through the execution of well-conducted clinical trials.^1^^(p9)^ The overall goal is to improve clinical outcomes for patients. Negative as well as positive findings of trials may influence practice; in particular, negative findings may inform health care professionals about which (potentially expensive) treatments do not work while also reducing costs and protecting patients from added treatment toxicity. Practice change may be best measured by the extent to which the results from clinical trials are used to support guideline care recommendations. We examined the association of network group trials with clinical practice guidelines and new drug approvals using study data from one of the largest NCTN groups.

## Methods

### Study Data

Study data were generated by the SWOG (formerly the Southwest Oncology Group) Cancer Research Network, a member of the NCTN.^3^ We identified randomized phase 3 SWOG trials designed to test the current standard of care (SOC) compared with experimental therapy using SWOG’s study database web portal (https://www.swog.org). Evaluable studies were those for which the primary end point was reported in a scientific journal from January 1, 1980, through June 30, 2017. Only trials led by SWOG or another collaborating NCI network group (ECOG-ACRIN [formerly the Eastern Cooperative Oncology Group and American College of Radiology Imaging Network], Alliance for Clinical Trials in Oncology, NRG Oncology [formerly the National Surgical Adjuvant Breast and Bowel Project, Radiation Therapy Oncology Group, and Gynecologic Oncology Group], or other) with SWOG centers and/or investigators contributing to patient accrual were included. Information on ethical review and informed consent of participants for each of the trials was included in their primary study reports. Institutional review board approval of this study was not required because this analysis used no patient-level data. Data were evaluated according to the Strengthening the Reporting of Observational Studies in Epidemiology (STROBE) reporting guideline.^4^

### Variable Definitions

To be considered practice influential (PI), a trial must have been associated with guideline care through its inclusion in clinical care guidelines or US Food and Drug Administration (FDA) new drug approvals in favor of a recommended treatment. We required more than 1 year since study publication to evaluate whether a trial was PI. Clinical care guidelines and FDA new indications were allowed as measures of practice influence because they represent different constructs that may not overlap; in particular, a negative trial finding may influence guideline care without leading to an FDA new indication.^5^ Importantly, a PI trial can only be considered to be associated with the practice of guideline clinical care because health care professionals do not always follow guideline recommendations.^6,7^ The categorization of PI trials was made independently by 2 authors (V.T.N. and R.V.), with disagreement resolved by a third author (J.M.U.).

A trial with positive findings was defined as one in which the experimental regimen achieved a statistically significant better result based on the study’s protocol-specified primary end point. All other studies—including those with negative or null findings—were categorized as negative. Trial designs were determined from protocol statistical sections or primary articles reporting the trial results. Categories of designs (superiority vs noninferiority vs equivalency) were determined using standard criteria.^8^

### Data Abstraction to Identify PI Trials

**Figure 1.**
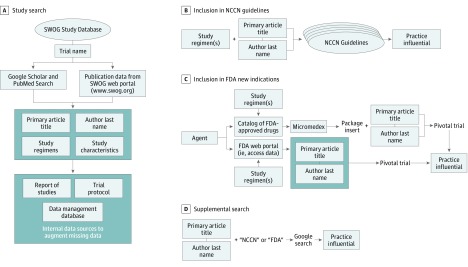
Search Algorithm to Identify Practice-Influential Phase 3 Cancer Clinical Treatment Trials FDA indicates US Food and Drug Administration; NCCN, National Comprehensive Cancer Network.

The primary articles for each study—those reporting the results of the analysis of the primary protocol-specified end point by randomized arm—were identified through SWOG’s publication database or manually using PubMed or Google Scholar. Primary article title, study regimens (agent, dose, administration, and sequence), and primary author last name were abstracted to use as search indices; study characteristics were also obtained (Figure 1A). Missing data for any study characteristics were augmented using internal information sources, including SWOG’s clinical data management database, trial protocols, and the regularly updated SWOG formal Report of Studies.

We used the National Comprehensive Cancer Network (NCCN) clinical practice guidelines from their inception in 1996 until 2019 to assess changes to guideline care (Figure 1B).^9^ The NCCN guidelines are comprehensive and continuously updated and apply to 97% of cancers affecting US patients.^10^ Each NCCN guideline version includes updates from prior versions, clinical charts representing primary treatment pathways and therapies, principles of treatment, and a discussion. We searched each cancer site-specific guideline using the search indices. If the trial provided evidence for the NCCN guideline recommendation (regardless of whether the finding was positive or negative) as cited in the clinical charts or principles of treatment sections, the trial was considered PI. For negative studies in particular, a PI trial was one for which the clinical charts or treatment sections indicated that the trial results reaffirmed SOC or, based on secondary evidence in the trial, suggested acceptable alternatives to SOC.

We also examined whether a trial led to an FDA new drug indication (Figure 1C). We constructed a comprehensive catalog of FDA-approved anticancer drugs based on multiple publicly available sources, including the FDA portal.^11,12^ If the trial’s experimental agent was included in this catalog, we obtained package inserts for the agent using the Micromedex drug information portal.^13^ As a secondary examination, we input each agent name into the FDA portal and traced the package insert or the FDA approval documentation that discussed the pivotal trial.^14^ If the drug was cited as pivotal in the package inserts or the FDA approval documentation, the associated trial was deemed to have resulted in an FDA new drug approval and was categorized as PI.

For trials not already classified as PI, we conducted a manual search using the Google search engine by using the combination of the first author’s last name and the title of the primary report, along with *Food and Drug Administration*, *FDA*, *National Comprehensive Cancer Network*, or *NCCN* as key words (Figure 1D). The same criteria were used to define a PI trial with respect to NCCN guidelines or FDA new drug approvals as noted earlier.

### Assessment of Costs

To estimate the total costs of conducting the trials, we obtained NCTN funding data from the National Institutes of Health’s Research Portfolio Online Reporting Tools by fiscal year starting in 1985.^15^ We also included funding to support early-stage trials (phases 1 and 2) as well as grants for statistical centers and member institutions supporting the conduct of NCTN trials. Nontreatment trial, biospecimen, and specific database project grants were excluded. Funding estimates were inflated to constant 2017 US dollars based on the Consumer Price Index.^16^ Funding for years 1980 through 1984, which was not available, was assumed to contribute the same amount in 2017 dollars as the subsequent 5-year period from 1985 through 1989.

### Statistical Analysis

Data were analyzed from June 15, 2018, to March 29, 2019. We reported the proportion of PI trials among all evaluable studies. Differences in rates of PI trials were compared between levels of study characteristics using χ^2^ tests (uncorrected) or Fisher exact tests where any cell counts were less than 5 for categorical variables and 2-sample *t* tests for continuous variables. Statistical significance was specified as 2-sided α ≤ .05. We used SAS, version 9.4 (SAS Institute Inc) and R, version 3.5.1 (R Project for Statistical Computing) to conduct the analyses.^17^

Total funding to conduct the trials was calculated as the total estimated funding for each network group, weighted by the estimated proportion that the number of trials led by each group represented of the group’s entire phase 3 trial portfolio. For SWOG, the number of phase 3 trials in the entire portfolio was identified from the study database. For other network groups, the number of phase 3 trials conducted during the period was estimated from ClinicalTrials.gov. Mean funding per trial for studies led by other groups (IBCSG [International Breast Cancer Study Group], EORTC [European Organisation for Research and Treatment of Cancer], etc) was assumed to be the mean amount among trials led by SWOG, Alliance, ECOG-ACRIN, and NRG. Total costs per completed trial, per PI study, and per FDA new drug approval were calculated.

## Results

### Study Design Characteristics

**Figure 2.**
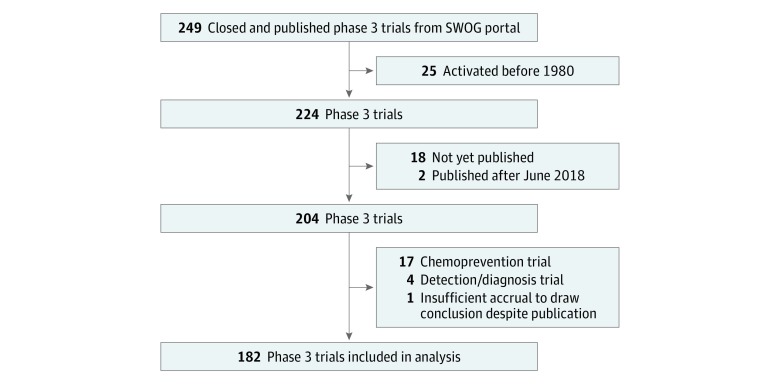
Trial Selection Flow Diagram

Two hundred forty-nine phase 3 clinical trials from the SWOG publication portal were identified as closed and published (Figure 2). One hundred eighty-two trials, including 148 028 patients, met the inclusion criteria. Ninety-four trials (51.6%) were led by SWOG and 88 (48.4%) by other groups. Accrual by group included 58 281 patients (39.4%) by SWOG, 22 028 (14.9%) by Alliance, 26 249 (17.7%) by ECOG-ACRIN, 12 051 (8.1%) by NRG, and 29 419 (19.9%) by other groups.

One hundred thirty-one trials (72.0%) included intergroup participation (Table 1). The most commonly investigated cancers included breast (30 [16.5%]), genitourinary (26 [14.3%]), lung (23 [12.6%]), and gastrointestinal tract (22 [12.1%]) cancers. Superiority designs accounted for 176 trials (96.7%). Advanced disease trials accounted for 120 studies (65.9%). Nearly all trials included systemic therapy as one of the treatments on any arm (177 [97.3%]). Median total accrual was 463 patients per trial (range, 60-7576); mean (SD) total accrual, 814 (1087) patients per trial.

**Table 1.** Characteristics of Phase 3 Cancer Treatment Clinical Trials at SWOG

Characteristic	No. (%) of Trials		*P* Value^b^
	All (n = 182)	Practice Influential (n = 82)^a^	
Cancer type			
Brain	7 (3.8)	0	.02
Breast	30 (16.5)	7 (23.3)	
Gastrointestinal tract	22 (12.1)	13 (59.1)	
Genitourinary	26 (14.3)	16 (61.5)	
Gynecologic	11 (6.0)	5 (45.5)	
Head and neck	7 (3.8)	5 (71.4)	
Leukemia	17 (9.3)	6 (35.3)	
Lung	23 (12.6)	9 (39.1)	
Lymphoma	12 (6.6)	6 (50.0)	
Melanoma	12 (6.6)	6 (50.0)	
Myeloma	8 (4.4)	4 (50.0)	
Others	7 (3.8)	5 (71.4)	
Disease setting			
Adjuvant	62 (34.1)	27 (43.5)	.77
Advanced	120 (65.9)	55 (45.8)	
Design type			
Superiority	176 (96.7)	77 (43.8)	.09
Equivalence or noninferiority	6 (3.3)	5 (83.3)	
End point type			
Overall survival	39 (21.4)	19 (48.7)	.80
Multiple, including overall survival	131 (72.0)	57 (43.5)	
Other	12 (6.6)	6 (50.0)	
No. of intervention arms			
2	119 (65.4)	53 (44.5)	.85
>2	63 (34.6)	29 (46.0)	
Intervention^c^			
Systemic therapy	177 (97.3)	80 (45.2)	>.99
Biological therapy	23 (12.6)	10 (43.5)	.87
Surgery	20 (11.0)	10 (50.0)	.64
Radiotherapy	43 (23.6)	22 (51.2)	.36
Transplant	13 (7.1)	5 (38.5)	.62
Blinded treatment	8 (4.4)	0	.009
Total accrual^d^			
Above the median	91 (50.0)	38 (41.8)	.37
Below the median	91 (50.0)	44 (48.4)	
Trial result			
Positive	65 (35.7)	47 (72.3)	<.001
Null or negative	117 (64.3)	35 (29.9)	
Intergroup trial			
Yes	131 (72.0)	68 (51.9)	.003
No	51 (28.0)	14 (27.5)	
Decade of trial completion			
1980-1989	29 (15.9)	9 (31.0)	.01
1990-1999	85 (46.7)	35 (41.2)	
2000-2009	55 (30.2)	33 (60.0)	
2010 and later	13 (7.1)	5 (38.5)	

Abbreviation: SWOG, (formerly) Southwest Oncology Group.

^a^ Calculated as percentage of all trials with the characteristic.

^b^ Calculated as difference in rates of PI trials between study characteristics of PI and non-PI trials using χ^2^ tests or Fisher exact test where any cell counts were less than 5 for categorical variables and 2-sample *t* tests for continuous variables.

^c^ Categories are not mutually exclusive.

^d^ Median number of patients was 463 (range, 60-7576).

### Overall Rate of PI Trials

**Figure 3.**
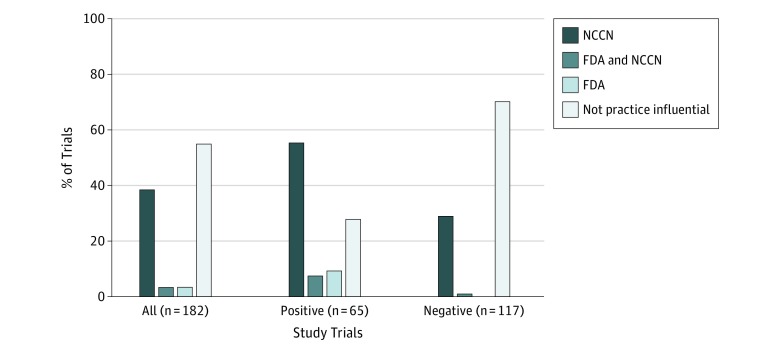
Disposition of Practice-Influential Trials Practice influence is determined by the trial’s influence on National Comprehensive Cancer Network (NCCN) guidelines, US Food and Drug Administration (FDA) new drug approvals, or both. Other trials were deemed not practice influential.

Overall, 82 of 182 trials (45.1%; 95% CI, 37.7%-52.6%) were PI (eTable 1 in the Supplement). Seventy trials (38.5%) influenced NCCN guidelines only; 6 (3.3%), FDA indications only; and 6 (3.3%), both (Figure 3).

Rates of PI trials differed among cancer types (*P* = .02), with lower rates observed among brain (0 of 7) and breast (7 of 30 [23.3%]) cancer trials and higher rates among head and neck (5 of 7 [71.4%]), genitourinary (16 of 26 [61.5%]), and gastrointestinal tract (13 of 22 [59.1%]) cancer trials (Table 1). Rates of PI trials were also higher among intergroup trials (68 of 131 [5.19%]; *P* = .003) and lower among blinded treatment trials (0 of 8; *P* = .009) and differed over time (*P* = .01), with the highest rate from 2000 to 2009 (33 of 55 [60.0%]).

Fourteen trials were cited in NCCN guidelines but were determined not to be PI because they did not support the recommended treatment (eTable 2 in the Supplement). Two trials (S8216 and S0106) (eTable 1 in the Supplement) were identified as PI through the manual search using Google Scholar (Figure 1D).^18,19^

### Rate of PI Trials Among Positive and Negative Findings

Among trials with positive findings, 47 of 65 (72.3%; 95% CI, 59.8%-82.7%) were PI, including 36 (55.4%) that influenced NCCN guidelines only, 5 (7.7%) that influenced FDA new drug indications only, and 6 (9.2%) that influenced both (Figure 3). Nine (4.9%) of the 11 trials (6.0%) that supported new FDA indications represented entirely new indications (rather than indications of previously approved regimens in other cancers) for single drugs (n = 7) or for drug combinations (n = 2) (eTable 3 in the Supplement).

Among trials with negative findings, 35 of 117 (29.9%; 95% CI, 21.8%-39.1%) were PI. Thirty-four trials (29.1%) influenced NCCN guidelines and 1 (0.9%) influenced the FDA to remove a listed indication.^20^ Overall, 35 of 82 PI trials (42.7%) were based on negative findings. Nearly half the studies with negative findings (17 of 35 [48.6%]) reaffirmed SOC over experimental therapy (eTable 1 in the Supplement). Among the remaining studies with negative findings, 15 provided sufficient evidence to suggest acceptable alternatives for SOC, and 3 influenced guidelines for other reasons.

### Additional Analyses

Early studies may have been less likely to be identified as PI because the NCCN guidelines only became available in 1996. However, among the 42 trials published before 1996, 16 trials were PI (38.1%); among the 140 trials published in 1996 or after, 66 were PI (47.1%; *P* = .39).

An additional 5 trials were identified as potentially PI based on the manual Google search (Figure 1D and eTable 4 in the Supplement) but did not meet the formal criteria of inclusion within NCCN or FDA materials. With these studies included as PI, the overall rate of PI trials was 87 of 182 (47.8%; 95% CI, 40.4%-55.3%).

The median number of additional studies cited as influential in NCCN guidelines or FDA package inserts—in addition to the SWOG study—was 1 (range, 0-14). The SWOG trial was the only cited study for 23 of the 82 PI trials (28.0%) and was 1 of 2 cited studies for 28 of the 82 PI trials (34.1%). The median number of additional studies cited did not differ between positive vs negative findings (median, 1; range, 0-14; *P* = .69).

### Cost per Trial

Total federal investment supporting the set of analyzed trials was estimated to be $1.36 billion in 2017 dollars. Given 182 total trials, this finding suggests the mean costs were $7.5 million per completed phase 3 trial (PI or non-PI), $16.6 million per PI trial, and $123.6 million per FDA new drug approval.

## Discussion

This study is the first, to our knowledge, to comprehensively examine the practice influence of NCTN-sponsored clinical trials for cancer using objective criteria. Nearly half (45.1%) of the trials we examined were found to be associated with guideline care through their inclusion in clinical care guidelines or FDA new drug indications, including many studies with negative findings, illustrating the value of well-conducted trials even when they do not demonstrate positive results. The investment cost for each PI trial was $16.6 million.

A few studies in the United Kingdom used physician surveys to show that multicenter randomized clinical trials influence clinical practice, with the effect extending beyond the participating physicians.^21,22^ Other studies have examined the research underlying cancer clinical practice guidelines across various countries.^23,24,25^ We found no evidence in the literature of studies that explicitly estimated how often primary clinical trial results are associated with guideline care.

The results in Table 1 are limited by small numbers but illuminate potentially informative associations. The high proportion of PI trials in head and neck cancers was due partly to 3 trials^26,27,28^ conducted in the 1990s showing that multimodality therapy (chemotherapy and radiotherapy) provided superior outcomes compared with radiotherapy alone, a strategy still commonly used today. This multimodality approach was also successful for trials across different cancer settings, in part accounting for the higher rate of PI trials from 2000 to 2009.^29,30^ The higher rate of PI trials in genitourinary cancers was associated with efforts to improve on androgen deprivation therapy for prostate cancer by including antiandrogrens,^31^ by modulating timing of androgen deprivation therapy administration,^32^ or more recently by adding other systemic therapy or radiotherapy.^33,34^ In bladder cancer, the discovery of bacillus Calmette-Guérin, a vaccine and early immunotherapeutic approach to the treatment of bladder cancer, was established in a series of trials showing its superiority over other SOCs.^18,35,36^ The lower rate of PI trials in breast cancer is somewhat misleading because 4 other trials identified in our supplemental search^37,38,39,40^ provided evidence against the use of transplant therapies for metastatic disease but did not meet our end point criteria.

Trials conducted by intergroups rather than single network groups were more likely to be PI. The collaboration of multiple network groups on a trial could reflect the importance of the original research question and the increased potential for the results to guide practice. Future research establishing a prediction model based on modifiable factors that reliably predict PI trial findings would represent a powerful use of study databases of the kind used in this analysis to influence policy.^41^

The success rate of pharmaceutical company phase 3 FDA indication studies has been examined extensively. DiMasi and colleagues^42^ used data from the largest 50 pharmaceutical companies for drug evaluations from 1993 through 2004 and found that about half of phase 3 trials were submitted for indication. Kola and Landis^43^ examined data from 10 large pharmaceutical companies from 1991 through 2000 and found the overall success rate from phase 3 trials through registration of about 28%. Hay and colleagues^44^ examined 835 drug developers from 2003 through 2011 to estimate an overall success probability from phase 3 trials in oncology of 37%. Together, approximately 35% of oncology drugs from phase 3 trials receive an FDA new drug approval in the pharmaceutical setting.^45^

Overall, 6.0% of phase 3 trials in this study led to a new FDA indication, including 4.9% leading to entirely new indications. These rates are lower than those observed in pharmaceutical company studies. However, the objective of NCTN trials is rarely to register a new drug indication. As noted by the Institute of Medicine, the primary focus of pharmaceutical company–sponsored trials is “to develop novel therapeutic agents and gain FDA approval for clinical use,”^1^^(p5)^ whereas the publicly funded NCTN clinical trials address research questions that are “important to patients but less likely to be top priorities of industry.”^1^^(p1)^ Thus, the focus of the NCTN’s groups is placed on trials that compare the effectiveness of treatment options already in use, that combine novel therapies developed by different companies, that develop therapies for rare diseases, that examine different doses and durations of existing therapies, or that test therapeutic approaches that combine multiple treatment modalities.^1^ Therefore, the rate at which network group clinical trials result in new drug indications is expected to be lower.

For pharmaceutical companies, the investment costs for each drug approval are high.^42,45,46,47,48,49,50,51,52,53^ One frequently cited statistic indicates a requirement of $1.1 billion (in 2017 US dollars).^48^ Many studies suggest the cost is even higher,^42,45,47,49,50,51,52^ especially for cancer drugs.^46^ Indeed, the mean cost estimate for a single new drug indication in 2017 dollars across 10 different studies^42,45,46,47,48,49,50,51,52,53^ we reviewed was $1.73 billion, more than 100 times greater than the federal investment cost per PI trial (Table 2). This comparison is imperfect because the pharmaceutical research process supports human trials and initial drug discovery, and the regulatory oversight for early-phase trials is more costly. Although our estimate of costs for NCTN trials also included those supporting early-stage (phases 1 and 2) trials, these trials are not often as closely tied to the eventual phase 3 trial and are not as numerous. Further, federal dollars invested in network group clinical trials frequently do not cover all of the capitation and institutional costs related to trial participation.^54^ For these reasons, network groups sometimes explicitly collaborate with industry to conduct trials, providing the design, conduct, and analysis of clinical trials, whereas industry may provide support for per-case costs at trial sites and the costs of nonstandard patient care and ancillary studies.^55^ Nonetheless, the observation highlights the value of the NCI’s network cancer research groups.

**Table 2.** Cost Estimates for New Drug Approval in the Pharmaceutical Industry^a^

Source	Study Period	Cost, US$	
		Estimate (Year)	Inflation Adjusted to 2017^b^
Adams and Brantner,^46^ 2006	1989-2002	868 million (2000)	1.23 billion
Adams and Brantner,^47^ 2010	1985-2001	1.2 billion (2000)	1.70 billion
DiMasi et al,^48^ 2003	1983-1994	802 million (2000)	1.14 billion
DiMasi et al,^42^ 2010	1990-2003	1.2 billion (2005)	1.50 billion
DiMasi et al,^45^ 2016	2005-2013	2.588 billion (2013)	2.71 billion
Gilbert et al,^49^ 2003	2000-2002	1.7 billion (20003)	2.26 billion
Mestre-Ferrandiz et al,^50^ 2012	1997-1999	1.5 billion (2011)	1.63 billion
O’Hagan and Farkas,^51^ 2009	2009	2.2 billion (2009)	2.50 billion
Paul et al,^52^ 2010	2007	1.8 billion (2008)	2.00 billion
Prasad and Mailankody,^53^ 2017	2006-2015	658 million	658 million

^a^ Adapted from DiMasi et al.^45^

^b^ Inflation adjusted to 2017 dollars using Consumer Price Index tables.^16^ Mean inflation-adjusted estimate was $1.73 billion.

One striking feature of our results is the number of negative trials found to influence guideline care. This factor is important because negative trials are generally perceived as failures. However, the true underlying objective of a well-conducted trial is to reduce uncertainty about the efficacy of a new treatment rather than to achieve any particular outcome. This notion is important given the history of exciting new therapeutic approaches found to be ineffective or outright harmful when tested in comparative clinical trials. In the 1990s, autologous bone marrow transplants for breast cancer were thought to represent an important alternative and potentially curable avenue for women with metastatic disease, supported by the exciting results of early-stage, noncontrolled trials.^56^ However, multiple randomized phase 3 trials—conducted even as transplants were already in use for many patients with breast cancer—found that transplants were expensive, resulted in consistently more treatment fatalities vs conventional chemotherapy, and showed no meaningful evidence of clinical benefit.^37,38,39,40^ Since the publication of these studies, transplant therapy in this setting has been largely abandoned.^57,58,59^ In another example, after the FDA approved gemtuzumab ozogamicin through the accelerated approval program for patients with acute myeloid leukemia, the confirmatory trial S0106 demonstrated increased deaths and no advantage in survival end points associated with this agent, leading to a voluntary postmarket withdrawal of gemtuzumab in 2010.^19,60^ Recently, this agent was newly approved by the FDA but only at a lower dose, a different schedule, and in the targeted setting of cases expressing the CD33 antigen.^20^

### Limitations

Our study was limited to the evaluation of trials directed or contributed to by a single large network group. This criterion could limit the generalizability of the overall estimate of PI trials, although importantly, other network groups led or contributed to almost 3 of every 4 trials. Also, our estimate may be biased high if the rate of intergroup trials was not representative because intergroup trials were more likely to influence practice. Further, although our determination of PI trials relied in part on NCCN guidelines, which are widely used by payers and health care professionals, other compendia may have generated a different set of estimates. Moreover, the identification of a PI trial may be subject to the frequency with which NCCN guidelines are updated for selected cancers, especially more recent trials and those conducted in less common cancers. Although in some instances a SWOG PI trial was solely responsible for influencing guideline care, in others, it was only 1 of multiple studies, suggesting contributions from trials not included in our study sample.

## Conclusions

Our findings show that nearly half of all randomized phase 3 trials conducted by one of the NCI’s large network clinical trial groups were associated with guideline care and new drug approvals. This rate exceeds the rate of phase 3 trials with positive findings (generally estimated to be 25%-30%) owing to the fact that many trials with negative findings—which are often considered scientific failures—also influence guideline care, especially by identifying what treatments should not be used. Thus, the true value of a well-conducted, controlled randomized clinical trial for cancer treatment is in guiding the cancer clinical treatment community about the best treatment choice regardless of whether the best choice is a new, trial-proven experimental therapy or the existing SOC. Moreover, NCTN trials also benefit research through data sharing and secondary analyses using the clinical study databases, which provide important insights into current treatments and hypotheses for future trials. Finally, the amount invested by federal funders to provide this valuable evidence was modest. The NCTN program contributes clinically meaningful, cost-efficient evidence to guide patient care.

## Notes

Supplement. **eTable 1.** Description and Key Findings of the 82 Practice-Influential Phase 3 Cancer Treatment Clinical Trials at SWOG **eTable 2.** NCCN-Cited Phase 3 Trials Determined Not to Be Practice Influential **eTable 3.** Trials That Influenced FDA New Indications **eTable 4.** Description and Key Findings of the 5 Potential Practice Influential Cancer Treatment Phase 3 Clinical Trials Without NCCN Endorsement and FDA Indication
