# Supplementary material for: Association of National Cancer Institute–Sponsored Clinical Trial Network Group Studies With Guideline Care and New Drug Indications
Source: JAMA Netw Open. 2019 Sep 4;2(9):e1910593. doi: 10.1001/jamanetworkopen.2019.10593 (PMC6727679; doi:10.1001/jamanetworkopen.2019.10593)
Supplement: Supplement. — eTable 1. Description and Key Findings of the 82 Practice-Influential Phase 3 Cancer Treatment Clinical Trials at SWOG eTable 2. NCCN-Cited Phase 3 Trials Determined Not to Be Practice Influential eTable 3. Trials That Influenced FDA New Indications eTable 4. Description and Key Findings of the 5 Potential Practice Influential Cancer Treatment Phase 3 Clinical Trials Without NCCN Endorsement and FDA Indication [file jamanetwopen-2-e1910593-s001.pdf]

Supplementary Online Content

Unger JM, Nghiem VT, Hershman DL, Vaidya R, LeBlanc M, Blanke CD. Association of National Cancer Institute–sponsored Clinical Trial Network group studies with guideline care and new drug indications. *JAMA Netw Open.* 2019;2(9):e1910593. doi:10.1001/jamanetworkopen.2019.10593

eTable 1. Description and Key Findings of the 82 Practice-Influential Phase 3 Cancer Treatment Clinical Trials at SWOG

eTable 2. NCCN-Cited Phase 3 Trials Determined Not to Be Practice Influential

eTable 3. Trials That Influenced FDA New Indications

eTable 4. Description and Key Findings of the 5 Potential Practice Influential Cancer Treatment Phase 3 Clinical Trials Without NCCN Endorsement and FDA Indication

This supplementary material has been provided by the authors to give readers additional information about their work.

© 2019 Unger JM et al. *JAMA Network Open*.

eTable 1. Description and Key Findings of the 82 Practice-Influential Phase 3 Cancer Treatment Clinical Trials at SWOG

Study ID Trial end	Cancer type	Experimental v. standard arm	Primary findings: Experimental regimens had…	Interpretation of positive or negative findings	Reference from NCCN guidelines or *others*
**A. Positive trials (k=47)**					
S8600[1] (1991)	AML	High dose of Cytosine Arabinoside with Daunorubicin **v.** Standard dose	Better PFS and WFS	Supported NCCN treatment recommendation	v. 2.2018, p. AML-8, p. MS-30-32
S9034 (1995)	AML	High-dose Cytarabine **v.** Autologous or allogeneic bone marrow transplantation	Better OS	Supported NCCN treatment recommendation	v. 1.2003, p. MS-6
S9129 (1995)	AML	Induction with All-trans-retinoic acid **v.** Induction with Daunorubicin + Cytarabine	Better OS and DFS	Supported NCCN treatment recommendation	v. 1.2003, p. MS-3
C9710 (2005)	AML	Standard induction and consolidation therapy + Arsenic trioxide **v.** Standard induction and consolidation therapy	Better event-free survival and DFS	Supported NCCN treatment recommendation	v. 2.2018, p. AML-4, p. MS-13, p. MS-14
S8216[1,2] (1987)	Bladder	Bacillus Calmette-Guerin **v.** Adriamycin	Better time to failure, response, and DFS	Supported FDA new drug approval	*Seminars in Oncology 35(5) (2008) 545-552*
S8507[1] (1988)	Bladder	Maintenance with Bacillus Calmette-Guerin **v.** Observation	Better RFS and WFS	Supported NCCN treatment recommendation	v. 5.2018, p. BL-F 1 of 3
S8594 (1989)	Bladder	Cisplatin + Methotrexate, Vinblastine, Doxorubicin **v.** Cisplatin	Better response and PFS	Supported NCCN treatment recommendation	v. 1.2009, p. MS-18
S8795[1,2] (1992)	Bladder	Bacillus Calmette-Guerin **v.** Mitomycin C	Better RFS	Supported FDA new drug approval	*TICE® BCG package insert*
S8710[1] (1998)	Bladder	Methotrexate + Vinblastine + Doxorubicin + Cisplatin after radical cystectomy **v.** Cystectomy alone	Better medial survival	Supported NCCN treatment recommendation	v. 5.2018, p. BL-G 1 of 5
S9410 (1997)	Breast	Cyclophosphamide + Dose-varying Doxorubicin + Paclitaxel **v.** Cyclophosphamide + Doxorubicin	Better DFS and OS	Supported NCCN treatment recommendation	v. 4.2017, p. MS-41, p. MS-42 *BMC Medicine (2015) 13 (195): 1-13*
E1199 (2002)	Breast	Weekly Paclitaxel or Docetaxel **v.** Every 3 weeks Paclitaxel or Docetaxel	Better OS and DFS	Supported NCCN treatment recommendation	v. 1.2018, p. BINV-K 1 of 5
E2100 (2004)	Breast	Paclitaxel + Bevacizumab **v.** Paclitaxel	Better DFS	Supported FDA new drug approval and NCCN treatment recommendation	v. 3.2018, p. BINV-O 2 of 6, p. MS-62 *AVASTIN® package insert*
N9831 (2005)	Breast	Doxorubicin + Cyclophosphamide - Paclitaxel - Trastuzumab **v.** Doxorubicin + Cyclophosphamide - Paclitaxel	Better DFS	Supported FDA new drug approval and NCCN treatment recommendation	v. 1.2018, p. BINV-K 3 of 5, p. MS-31, p. MS-53 *HERCEPTIN® package insert*
IBCSG25 02 (2007)	Breast	Exemestane + GnRH agonist triptorelin **v.** Tamoxifen + Triptorelin	Reduced recurrence	Supported NCCN treatment recommendation	v. 1.2018, p. BINV-J, p. MS-36
S8695 (1990)	Cervical	RT + 5-FU + Cisplatin **v.** RT + Hydroxyurea	Better PFS and OS	Supported NCCN treatment recommendation	v. 1.2018, p. MS-8, p. MS-10, p. MS-11
S8797[1] (1996)	Cervical	Cisplatin-based chemotherapy + RT **v.** RT	Better PFS and OS	Supported NCCN treatment recommendation	v. 3.2019, p. MS-9, p. MS-14, p. MS-23

© 2019 Unger JM et al. *JAMA Network Open*.

Study ID Trial end	Cancer type	Experimental v. standard arm	Primary findings: Experimental regimens had…	Interpretation of positive or negative findings	Reference from NCCN guidelines or *others*
S8591[1,2] (1987)	Colon	Levamisole + Fluorouracil **v.** Observation	Better outcomes in recurrence and mortality rate	Supported FDA new drug approval	*CA Cancer J Clin (1997) 47: 243-256*
N9741 (2002)	Colorectal	Infused 5-FU + Leucovorin + Oxaliplatin **v.** Reduced-dose 5-FU + Leucovorin + Irinotecan	Better response, time to progression, and OS	Supported NCCN treatment recommendation	v. 3.2018, p. MS-5, p. MS-27
S8598 (1991)	Esophagus	5-FU + Cisplatin + RT **v.** RT	Better OS, better outcomes in recurrence and side effect	Supported NCCN treatment recommendation	v. 2.2018, p. MS-18
S9008[1] (1998)	Gastric	5-FU + Leucovorin + local-regional radiation after surgery **v.** Surgery alone	Better RFS and OS	Supported NCCN treatment recommendation	v. 2.2018, p. GAST-E, p. GAST-F 7 of 12
S8892[1] (1995)	HN	Cisplatin + 5-FU + radiotherapy **v.** Radiotherapy alone	Better PFS and OS	Supported NCCN treatment recommendation	v. 2.2018, p. CHEM-A 1 of 5, p. MS-26, p. MS-29, p. MS-30
S9059 (1999)	HN	RT + Cisplatin or RT + 5-FU + Cisplatin **v.** RT	Better OS	Supported NCCN treatment recommendation	v. 2.2018, p. CHEM-A 3 of 5, p. MS-24, p. MS-26, p. MS-36
S9515 (2000)	HN	Cisplatin + RT **v.** RT	Better DFS and fewer adverse events	Supported NCCN treatment recommendation	v. 2.2018, p. OR-A 2 of 2, p. ORPH-A 2 of 2, p. HYPO-A 2 of 2, p. GLOT-A 2 of 2, p. SUPRA-A 2 of 2, p. ADV-A 2 of 2, p. OCC-A 2 of 2, p. CHEM-A 1 of 5, p. MS-8, p. MS-43
S8691[1] (1991)	Leukemia	Pentostatin **v.** Interferon alfa - 2a	Better RR	Supported NCCN treatment recommendation	v. 2.2019, p. HCL-B, p. MS-3
S8393[1] (1992)	Melanoma	Excision 2cm **v.** Excision 4cm	Better outcomes (less need of skin grafting, shortened hospital stay)	Supported NCCN treatment recommendation	v. 3.2018, p. MS-16
S9111 (1995)	Melanoma	High-dose Interferon alfa-2b or low-dose Interferon alfa-2b **v.** Observation	Better OS	Positive, FDA indication, supporting treatment recommendation	v. 3.2018, p. MS-25 *INTRON© A package insert*
S9512 (1999)	Melanoma	GMK vaccine **v.** High-dose Interferon alfa-2b	Better RFS and OS	Supported NCCN treatment recommendation	v. 3.2018, p. MS-25
S0008[1] (2007)	Melanoma	Cisplatin + Vinblastine + Dacarbazine + Interferon alfa-2b **v.** High-dose Interferon alfa-2b	Better RFS	Supported NCCN treatment recommendation	v. 3.2018, p. ME-H 5 of 5, p. MS-25
S9210[1] (1998)	Myeloma	Alternate-day Prednisone 50mg **v.** 10mg	Better PFS and OS	Supported NCCN treatment recommendation	v. 1.2003, p. MYEL-C, p. MS-1
S0232[1] (2007)	Myeloma	Lenalidomide + high-dose Dexamethasone **v.** Standard-dose Dexamethasone	Better PFS and response	Positive, supporting the addition of the second agent to standard of care	v. 1.2019, p. MS-13
S0777[1] (2012)	Myeloma	Bortezomib + Lenalidomide + Dexamethasone **v.** Lenalidomide + Dexamethasone	Better PFS and OS	Supported NCCN treatment recommendation	v. 2.2019, p. MS-12, p. MS-15
S8736[1] (1995)	NHL	CHOP + RT **v.** CHOP	Better PFS and OS	Supported NCCN treatment recommendation	v. 2019, p. BCEL-A 1 of 2, p. BCEL-C 3 of 4

© 2019 Unger JM et al. *JAMA Network Open*.

Study ID Trial end	Cancer type	Experimental v. standard arm	Primary findings: Experimental regimens had…	Interpretation of positive or negative findings	Reference from NCCN guidelines or *others*
E4494 (2001)	NHL	Rituximab + CHOP **v.** CHOP	Better FFS	Supported NCCN treatment recommendation	v. 1.2019, p. MANT-A 4 of 4, p. MS-104
S9704[1] (2007)	NHL	1 time Induction + Autologous stem-cell transplantation **v.** 3 time Induction	Better PFS	Supported NCCN treatment recommendation	v. 2019, p. MS-103
S8991 (1992)	SCLC	Cisplatin + Etoposide + twice-daily RT **v.** Cisplatin + Etoposide + once-daily RT	Better OS	Supported NCCN treatment recommendation	v. 2.2018, p. SCL-E 1 of 3, p. SCL-F 1 of 3, p. MS-13
S8992 (1992)	NSCLC	Cisplatin + Vinblastine + RT **v.** Standard RT or Hyperfractionated RT	Better OS	Supported NCCN treatment recommendation	v. 2.2019, p. NSCLC-C 4 of 10
S9308[1,2] (1995)	NSCLC	Vinorelbine + Cisplatin **v.** Cisplatin	Better PFS and response	Supported FDA new drug approval	*NAVELBINE® package insert*
JBR10 (2001)	NSCLC	Vinorelbine + Cisplatin **v.** Observation	Better RFS and OS	Supported NCCN treatment recommendation	v. 5.2018, p. NSCL-D, p. MS-56
S8412[1] (1989)	Ovarian	Carboplatin + Cyclophosphamide **v.** Cisplatin + Cyclophosphamide	Better therapeutic index	Positive, FDA indication, supporting treatment recommendation	*Br J Cancer (1998) 78(11): 1479-1487*<br>*PARAPLATIN® package insert*
S9701[1] (2001)	Ovarian	Paclitaxel 12 months **v.** Paclitaxel 3 months	Better PFS	Supported NCCN treatment recommendation	v. 2.2018, p. MS-16
S8494[1,2] (1987)	Prostate	Leuprolide + Flutamide **v.** Leuprolide	Better PFS and OS	Supported FDA new drug approval	*EULEXIN® package insert*
S8793 (1993)	Prostate	Immediate hormonal therapy **v.** Observation	Better OS	Supported NCCN treatment recommendation	v. 4.2018, p. MS-27
S9916[1] (2003)	Prostate	Prednisone + Docetaxel **v.** Estramustine + Mitoxantrone	Better OS, time to progression and rate of prostate-specific antigen	Positive, FDA indication, supporting treatment recommendation	v. 4.2018, p. MS-28<br>*FDA medical review for application no. 203415Orig1s000*<br>*Rev in Urol (2007) Vol 9, Suppl 2: S13-8*
JPR3 (2005)	Prostate	RT + Androgen deprivation therapy **v.** Androgen deprivation therapy	Better OS	Supported NCCN treatment recommendation	v. 4.2018, p. MS-19, p. MS-28
E3805 (2012)	Prostate	Docetaxel + ADT **v.** ADT	Better OS	Supported NCCN treatment recommendation	v. 4.2018, p. MS-39, p. MS-51
S8949[1] (1998)	Renal	Nephrectomy + Interferon alfa - 2b **v.** Interferon alfa - 2b	Better median survival	Supported NCCN treatment recommendation	v. 2.2019, p. MS-10
S8896 (1990)	Rectal	Protracted venous infusion 5-FU **v.** Bolus 5-FU	Better OS, reduced tumor relapse and distant metastasis	Supported NCCN treatment recommendation	v. 3.2018, p. REC-D 1 of 2, p. MS-29
**B.1. Negative trials reaffirming standard of care (k=17)**					
S8514[1] (1989)	HN	Cisplatin + 5-FU or Carboplatin + 5-FU **v.** Methotrexate	Better response	Reaffirmed standard of care as single-agent therapy	v 2.2018, p. CHEM-A 2 of 5, p. MS-37, p. MS-38
S8516[1] (1991)	NHL	m-BACOD or ProMACE-CytaBOM or MACOP-B **v.** CHOP	No difference	Reaffirmed standard of care	v. 1.2019, p. MS-100

© 2019 Unger JM et al. *JAMA Network Open*.

Study ID Trial end	Cancer type	Experimental v. standard arm	Primary findings: Experimental regimens had…	Interpretation of positive or negative findings	Reference from NCCN guidelines or *others*
S8616[1] (1991)	Sarcoma	Ifosfamide + Mesna added to Doxorubicin + Dacarbazine **v.** Doxorubicin + Dacarbazine	Better response rate and better outcome in time to progression	Reaffirmed standard of care without the addition of Ifosfamide	v. 2.2018, p. SARC-F 3 of 6, p. MS-11
S0033[1] (2001)	Sarcoma	High-dose Imatinib Mesylate **v.** Standard dose	No difference	Reaffirmed low-dose Imatinib as initial standard dose	v. 1.2019, p. MS-31, *JNCCN (2007) Vol 5, Suppl 2: S1-31*
R9811 (2005)	Anal canal	Fluorouracil + Cisplatin + RT **v.** Fluorouracil + Mitomycin + RT	Worse survival and DFS	Reaffirmed standard of care	v. 2.2018, p. ANAL-B 1 of 2, p. ANAL-C 1 of 2, p. ANAL-C 2 of 2, p. MS-10, p. MS-13
30987 (2004)	Bladder	Paclitaxel + Cisplatin + Gemcitabine **v.** Cisplatin + Gemcitabine	Better response, better OS in subgroup	Reaffirmed standard of care without Paclitaxel	v. 5.2018, p. BL-G 2 of 5
C89803 (2001)	Colon	Irinotecan + 5-FU + Leucovorin **v.** 5-FU + Leucovorin	No difference	No recommendation of Irinotecan as treatment	v. 3.2018, p. MS-20
S8789 (1990)	Germ Cell	Etoposide + Carboplatin **v.** Etoposide + Cisplatin	No difference	Reaffirmed standard of care and suggested alternative treatment for specific patient group	v. 2.2018, p. MS-10
E1395 (2000)	HN	Cisplatin + 5-FU **v.** Cisplatin + Paclitaxel	No difference	Recommended standard arm (Cisplatin + Paclitaxel)	v. 2.2018, p. CHEM-A 2 of 5
S8952 (1995)	Hodgkin's Lymphoma	ABVD vs. MOPP/ABV	No difference	Recommended standard arm (ABVD)	v. 1.2010, p. HODG-B
E2496 (2006)	Hodgkin's Lymphoma	Doxorubicin + Bleomycin + Vinblastine + Dacarbazine **v.** Trimethoprim/Sulfamethoxazole + Ketoconazole + involved-field RT	No difference	Reaffirmed either ABVD or Stanford V as primary treatment	v. 3.2018, p. HODG-7, p. HODG-8, p. HODG-10, p. HODG-11, p. HODG-B 1 of 2, p. MS-14, p. MS-15
E2603 (2008)	Melanoma	Paclitaxel + Carboplatin + Sorafenib **v.** Paclitaxel + Carboplatin	No difference	Reaffirmed standard of care without sorafenib	v. 3.2018, p. ME-H 5 of 5, p. MS-54
G0182[3] (2004)	Ovarian	Carboplatin + Paclitaxel **v.** Carboplatin + Paclitaxel (different doses, schedules) **v.** Carboplatin + Methoxypolyethylene glycosylated liposomal Doxorubicin **v.** Carboplatin + Topotecan (different doses, schedules) **v.** Carboplatin + Gemcitabine + Carboplatin + Paclitaxel	No difference	Demonstrated no evidence that more than 6 cycles of combination chemotherapy are required for initial chemotherapy	v. 2.2018, p. MS-13
C9732 (2001)	SCLC	Etoposide + Cisplatin + Paclitaxel **v.** Etoposide + Cisplatin	No difference	Reaffirmed standard of care without Paclitaxel	v. 2.2018, p. SCL-E 1 of 3, p. MS-8
S8997 (1992)	Testicular	Cisplatin + Etoposide + Ifosfamide **v.** Cisplatin + Etoposide + Bleomycin	No difference	Reaffirmed standard of care	v. 2.2018, p. TEST-E, p. MS-16
S9346[1] (2008)	Prostate	Intermittent Bicalutamide Goserelin Acetate **v.** Continuous Bicalutamide Goserelin Acetate	No difference	Confirmed no evidence to determine intermittent ADT better than continuous ADT	v. 4.2018, p. MS-33
JPR7 (2005)	Prostate	Intermittent ADT **v.** Continuous ADT	No difference	Reaffirmed no evidence to determine intermittent ADT better than continuous ADT	v. 4.2018, p. MS-32
**B.2. Negative trials suggesting acceptable alternatives to standard of care (k=15)**					

© 2019 Unger JM et al. *JAMA Network Open*.

Study ID Trial end	Cancer type	Experimental v. standard arm	Primary findings: Experimental regimens had…	Interpretation of positive or negative findings	Reference from NCCN guidelines or *others*
S8692[1,2] (1995)	Breast	Goserelin **v.** Ovariectomy	No difference	FDA indication supporting Goserelin as an alternative treatment	*ZOLADEX® package insert*
S9304 (2000)	GI	Bolus FU + FU via protracted venous infusion vs. FU via protracted venous infusion only vs. Bolus FU + Leucovorin	No difference	Suggested alternate therapies for patients that cannot tolerate infusional FU or Capecitabine	v 3.2018, p. MS-19
S0518[1] (2012)	NET	Depot Octreotide + Interferon alfa-2b **v.** Depot Octreotide + Bevacizumab	No difference	Suggested experimental arm as acceptable alternative treatment	v. 2.2018, p. MS-15
S9321[1] (2000)	Myeloma	Hematopoietic cell-supported high-dose therapy **v.** Standard-dose therapy	No difference	Reaffirmed autologous stem-cell transplant as an option after primary induction therapy	v. 2.2016, p. MYEL-6, v. 3.2018, p. MS-21, v. 2.2019, p. MS-18, p. MS-22
S9252 (1996)	NSCLC	Postoperative therapy vs. Placebo	No difference	Suggested postoperative radiation with concurrent chemotherapy for medically fit patients	v. 5.2018, p. NSCL-C 2 of 10
S9509[1] (1998)	NSCLC	Paclitaxel + Carboplatin **v.** Vinorelbine + Cisplatin	Better toxicity and tolerability	Demonstrated that many platinum-doublet combinations yield similar objective response rates and survival	v. 5.2018, p. MS-31
S9900[1] (2004)	NSCLC	Post-surgery Paclitaxel + Carboplatin **v.** Surgery alone	No difference	Supported preoperative chemotherapy as a treatment option	v. 5.2018, p. MS-26
S8024[1] (1986)	Sarcoma	Continuous infusion therapy with Doxorubicin + Dacarbazine **v.** Bolus Doxorubicin + Dacarbazine	Better tolerance reduced toxicity	Supported combination therapy regardless of method of administration	v. 2.2018, p. SARC-F 1 of 6, p. MS-11
S9040 (1992)	Rectal	Infusional 5-FU + Levamisole or Infusional 5-FU + Leucovorin or Infusional 5-FU + Levamisole + Leucovorin **v.** bolus 5-FU alone	No difference	Supported recommendation of alternative therapies for patients intolerant to bolus 5-FU	v. 3.2018, p. REC-B 3 of 6, p. REC-D 1 of 2, p. MS-17
S8794[1] (1997)	Prostate	RT **v.** Observation	No difference in survival but better outcome in risk of prostate-specific antigen relapse and disease recurrence	Recommended adjuvant RT after radical prostatectomy as a management option	v. 4. 2018, p. MS-48
IBCSG24 02 (2010)	Breast	Tamoxifen + Ovarian suppression **v.** Exemestane + Ovarian suppression	Better outcomes for patient subgroup	Supported treatment recommendation for subgroups	v. 1.2018, p. MS-36
S8899 (1992)	Colon	Low-dose Leucovorin + 5-FU or High-dose Leucovorin + 5-FU or Low-dose Leucovorin + Levamisole + 5-FU **v.** Levamisole plus 5-FU	No difference in OS and PFS but low-dose and high-dose Leucovorin + 5-FU without Levamisole were better tolerated than usual care	Supported the removal of Levamisole from usual care	v. 3.2018, p. COL-G 2 of 2, MS-14
E3695 (2002)	Melanoma	Cisplatin + Vinblastine + Dacarbazine + Interleukin-2 + Interferon alfa-2b **v.** Cisplatin + Vinblastine + Dacarbazine	No difference	Recommended combination chemotherapy (dacarbazine or temozolomide-based including cisplatin and vinblastine) with or without	v. 1.2019, p. MS-53 *JNCCN (2009) 7(3): 251-275*

© 2019 Unger JM et al. *JAMA Network Open*.

Study ID Trial end	Cancer type	Experimental v. standard arm	Primary findings: Experimental regimens had…	Interpretation of positive or negative findings	Reference from NCCN guidelines or *others*
				interleukin-2 and interferon-alfa-2b as one option for systemic therapy	
S8592 (1988)	MDS	Low-dose Cytarabine **v.** Supportive therapy	No difference	Supported low-dose cytarabine therapy	v. 1998, p. 71
R04 (2010)	Rectal	RT + Capecitabine +/- Oxaliplatin **v.** RT + 5-FU +/- Oxaliplatin	No difference	5-FU or Capecitabine produced similar outcomes. Addition of oxaliplatin added toxicity without improving outcomes	v. 3.2018, p. REC-D 1 of 2, p. MS-19
**B.3. Negatives trial influencing guideline care in other ways (k=3)**					
S0106[1] (2009)	Leukemia	Daunorubicin + Cytarabine + Gemtuzumab Ozogamicin **v.** Daunorubicin + Cytarabine	No difference	Gemtuzumab Ozogamicin withdrawn from the market after the FDA request under the accelerated approval program	*J Clin Oncol (2013) 121(24): 4854-60*
N0147 (2009)	GI	modified FOLFOX + Cetuximab **v.** modified FOLFOX	No difference	Changed adjuvant therapy recommendations for patients undergoing surgical resection from advanced disease regimens to those regimens used for stage III disease	v. 4.2018, p. MS-22 *NCCN guideline meeting 2011*
S9336 (2001)	NSCLC	Cisplatin + Etoposide + RT + Surgery **v.** Cisplatin + Etoposide	No difference	Demonstrated preoperative chemoradiotherapy is controversial	v. 5.2018, p. NSCL-B 2 of 4, p. NSCL-C 2 of 10, p. MS-20

ABVD, Doxorubicin, Bleomycin, Vinblastine, and Dacarbazine; ADT, Androgen deprivation therapy; CHOP, Cyclophosphamide, Doxorubicin, Vincristine and Prednisone; DFS, Disease-free survival; FU, Fluorouracil; m-BACOD, Methotrexate with Leucovorin rescue, Bleomycin, Doxorubicin, Cyclophosphamide, and Etoposide; MACOP-B, Methotrexate with Leucovorin rescue, Doxorubicin, Cyclophosphamide, Vincristine, Prednisone, and Bleomycin; MOPP/ABV, Mechlorethamine, Vincristine, Procarbazine, Prednisone, Doxorubicin, Bleomycin, and Vinblastine; PFS, Progression free-survival; ProMACE-CytaBOM, Prednisone, Doxorubicin, Cyclophosphamide, and Etoposide followed by Cytarabine, Bleomycin, Vincristine, and Methotrexate with Leucovorin rescue; OS, overall survival; RFS, Recurrence-free survival; RR, Response rate; WFS, Worsening-free survival.

[1] SWOG-led study

[2] FDA indication without NCCN endorsement

[3] Assessment of clinical impact of this trial involved extended communication with the NCCN medical writing team.

© 2019 Unger JM et al. *JAMA Network Open*.

eTable 2. NCCN-Cited Phase 3 Trials Determined Not to Be Practice Influential

Study No. (Trial end)	Cancer type	Experimental regimens vs. control arm	Primary findings: Experimental regimen had…	In NCCN guidelines, the trial…	Reference from NCCN guidelines
S0521 (2010)	AML	Tretinoin + Mercaptopurine + Methotrexate vs. Observation	No difference	Assisted the discussion on possible maintenance therapies but not leading to any clinical recommendation	v 2. 2018, p. MS-23
S9402 (2002)	Brain	Chemotherapy + RT vs. RT	No difference	Assisted the discussion on diagnosis but not leading any clinical treatment recommendation	v 1.2018, p. MS-7
S8697 (1991)	Breast	Continuous chemotherapy vs. Observation	No difference	Assisted the discussion on the possible therapies but not leading to any clinical recommendation	v 1.2018, p. MS-61
S8851 (1994)	Breast	CAF + Goserelin + Tamoxifen vs. CAF + Goserelin vs. CAF	Better outcome on time to recurrence	Assisted the discussion on possible therapies but not leading any clinical recommendation	v 1.2018, p. MS-34
S9332 (1995)	Breast	Doxorubicin + Paclitaxel vs. Doxorubicin vs. Paclitaxel	No difference	Assisted the discussion on possible therapies using single or multiple drugs but not leading to any clinical recommendation	v 1.2018, p. MS-61
JMA17 (2002)	Breast	Letrozole vs. Placebo	Better disease-free progression survival	Assisted in the clinical expert panel's discussion on possible therapies but not leading to any clinical recommendation	v 1.2018, p. MS-36, p. MS-38, p. MS-39
S0226 (2009)	Breast	Anastrozole + Fulvestrant vs. Anastrozole	Better overall survival, better profession-free survival, better hazard ratio for progression or death	Assisted the discussion but not leading to any clinical recommendation	v 1.2018, p. MS-58
N9841 (2003)	GI	FOLFOX4 vs. Irinotecan	No difference	Assisted the discussion on possible therapies but not leading to any clinical recommendation	v 3.2018, p. MS-47
S0205 (2006)	GI	Gemcitabine + Cetuximab vs. Gemcitabine	No difference	Assisted the discussion on investigational therapies but not leading to any clinical recommendation	v 3.2019, p. MS-18
E2805 (2010)	GU	Sunitinib + Sorafenib vs. Placebo	No difference	Assisted the discussion on an ununiform consensus on the clinical recommendation	v 3.2018, p. MS-6
E1697 (2010)	Melanoma	Interferon alpha-2b vs. Observation	No difference	Assisted the discussion on doses/schedules	v 3.2018, p. MS-24
CTN0102 (2007)	Myeloma	Allogeneic HSCT with non-myeloablative conditioning after autologous HSCT vs. tandem autologous HSCT	No difference	Informed investigational therapies but not leading to any clinical recommendation	v 1.2019, p. MS-22
S9216 (1996)	SCLC	CODE vs. CAV/EP	No difference	Assisted the discussion on the multidrug cyclic weekly regimens but not leading to any clinical recommendation	v 2.2018, p. MS-9
S0124 (2007)	SCLC	Cisplatin + Irinotecan vs. Cisplatin + Etoposide	No difference	Assisted the discussion on drug combinations but not leading to any clinical recommendation	v 2.2018, p. MS-8

CAF, Cyclophosphamide + Doxorubicin + Fluorouracil, CAV/EP, alternating Cyclophosphamide, Doxorubicin, Vincristine/Etoposide and Cisplatin, CODE, Cisplatin + Vincristine + Doxorubicin + Etoposide, HSCT, Haemopoietic stem-cell transplantation

© 2019 Unger JM et al. *JAMA Network Open*.

eTable 3. Trials That Influenced FDA New Indications

Study No. (Trial end)	Cancer type	Experimental regimens vs. (usual care/comparators)	Primary findings: Experimental regimens had…	Positive/negative findings, evidence for	Reference from NCCN guidelines or *others*
**A. Positive trials**					
S8216[a] (1987) SWOG-led	Bladder	Bacillus Calmette-Guerin (Adriamycin)	Better time to failure, response, and probability of being disease-free	Positive, FDA indication<br>Entire new indication	*Seminars in Oncology 35(5) (2008) 545-552*
S8795[a] (1992) SWOG-led	Bladder	Bacillus Calmette-Guerin (Mitomycin C)	Better recurrence-free survival	Positive, FDA indication<br>Repeat evidence	*TICE® BCG package insert*
E2100 (2004)	Breast	Paclitaxel + Bevacizumab (Paclitaxel)	Better disease-free survival	Positive, FDA indication, supporting treatment recommendation<br>Drug combination as entire new indication<br>AVASTIN (Bevacizumab) not a new indication, first FDA approved 1998	v. 3.2018, p. BINV-O 2 of 6, p. MS-62<br>*AVASTIN® package insert*
N9831 (2005)	Breast	Doxorubicin + Cyclophosphamide - Paclitaxel - Trastuzumab (Doxorubicin + Cyclophosphamide - Paclitaxel)	Better disease-free survival	Positive, FDA indication, supporting treatment recommendation<br>Drug combination as entire new indication<br>Earlier, Herceptin was already part of another drug combination as the first new indication for BrCa.	v. 1.2018, p. BINV-K 3 of 5, p. MS-31, p. MS-53<br>*HERCEPTIN® package insert*
S8591[a] (1987) SWOG-led	Colon	Levamisole + Fluorouracil (Observation)	Better outcomes in recurrence and mortality rate	Positive, FDA indication<br>Entire new indication	*CA Cancer J Clin (1997) 47: 243-256*
S9111 (1995)	Melanoma	High-dose Interferon alfa-2b or low-dose Interferon alfa-2b (Observation)	Better overall survival	Positive, FDA indication, supporting treatment recommendation<br>Just change the dose	v. 3.2018, p. MS-25<br>*INTRON© A package insert*
S9308[a] (1995) SWOG-led	NSCLC	Vinorelbine + Cisplatin (Cisplatin)	Better response and progression-free survival	Positive, FDA indication<br>Entire new indication	*NAVELBINE® package insert*
S8412 (1989) SWOG-led	Ovarian	Carboplatin + Cyclophosphamide (Cisplatin + Cyclophosphamide)	Better therapeutic index	Positive, FDA indication, supporting treatment recommendation<br>Entire new indication	*Br J Cancer (1998) 78(11): 1479-1487*<br>*PARAPLATIN® package insert*
S8494[a] (1987) SWOG-led	Prostate	Leuprolide + Flutamide (Leuprolide)	Better progression-free survival and median survival	Positive, FDA indication<br>Entire new indication	*EULEXIN® package insert*
S9916 (2003) SWOG-led	Prostate	Prednisone + Docetaxel (Estramustine + Mitoxantrone)	Better overall survival, time to progression and rate of prostate-specific antigen	Positive, FDA indication, supporting treatment recommendation<br>Entire new indication	v. 4.2018, p. MS-28<br>*FDA medical review for application no. 203415Orig1s000*<br>*Rev in Urol (2007) Vol 9, Suppl 2: S13-8*

© 2019 Unger JM et al. *JAMA Network Open*.

**Study No. (Trial end)**	**Cancer type**	**Experimental regimens vs. (usual care/comparators)**	**Primary findings: Experimental regimens had…**	**Positive/negative findings, evidence for**	**Reference from NCCN guidelines or *others***
S8692[a] (1995) SWOG-led	Breast	Goserelin (Ovariectomy)	No difference	Negative, FDA indication, supporting goserelin as an alternate treatment Entire new indication	*ZOLADEX® package insert*

[a] No concurrent NCCN endorsement

© 2019 Unger JM et al. *JAMA Network Open*.

eTable 4. Description and Key Findings of the 5 Potential Practice Influential Cancer Treatment Phase 3 Clinical Trials Without NCCN Endorsement and FDA Indication

**Study No. (Trial end)**	**Cancer type**	**Experimental regimens vs. (usual care/comparators)**	**Primary findings: Experimental regimen had…**	**Interpretation of negative findings**	**Reference from NCCN guidelines or *others***
S8326[1] (1992)	AML	High-dose Cytarabine + Mitoxantrone **v.** High-dose Cytarabine	No difference	Reaffirmed standard of care	*Leukemia Research 2015, 39(2015):945-949*
S9412 (1997)	Breast	High-dose chemotherapy + stem-cell transplantation **v.** Cyclophosphamide + Methotrexate + Fluorouracil	No difference	Rejected transplantation therapy	*False hope: Bone marrow transplantation for breast cancer (2007), p. 249*<br>*Health Affairs 2001, 20(5):101-17*
S9061 (1998)	Breast	High-dose chemotherapy + stem-cell transplantation **v.** Conventional chemotherapy	No difference	No evidence of survival superiority when adding high-dose chemotherapy and autologous hematopoietic stem-cell transplantation to standard adjuvant chemotherapy	*False hope: Bone marrow transplantation for breast cancer (2007), p. 249*
S9114 (1998)	Breast	High-dose Cyclophosphamide + Cisplatin + Carmustine + Bone marrow transplant + Peripheral-blood progenitor cell support **v.** Intermediate-dose Cyclophosphamide + Cisplatin + Carmustine + GCSF	No difference	No evidence of the superiority of the experimental arm with stem-cell support compared with standard arm without stem-cell support	*False hope: Bone marrow transplantation for breast cancer (2007), p. 249*
S9623[1] (2001)	Breast	Doxorubicin + Cyclophosphamide followed by high-dose chemotherapy **v.** Sequential Doxorubicin + Paclitaxel, Cyclophosphamide + GCSF	No difference	No evidence that adjuvant high dose chemotherapy with autologous hematopoietic progenitor cell support improved outcomes	*False hope: Bone marrow transplantation for breast cancer (2007), p. 250*

GCSF = Granulocyte colony stimulating factor

© 2019 Unger JM et al. *JAMA Network Open*.
